# How parents experience their adolescent’s disclosure of previous sexual abuse: a qualitative study

**DOI:** 10.1186/s12888-023-05410-7

**Published:** 2023-12-06

**Authors:** Philippe Mauny, Sélim Benjamin Guessoum, Marie Rose Moro, Rahmeth Radjack, Émilie Carretier

**Affiliations:** 1 APHP, Cochin Hospital, Maison de Solenn, Paris, F-75014 France; 2 Paris Est Créteil University, Créteil, France; 3 Paris-Saclay University, UVSQ, Inserm, CESP, Team DevPsy, Villejuif, F-94807 France; 4 Paris Cité University, PCPP, Boulogne-Billancourt, F-92100 France; 5 Pôle Psychiatrie de l’Adolescent, Institut MGEN, La Verrière, France

**Keywords:** Child Sexual Abuse, Disclosure, Adolescents, Parents, Qualitative study

## Abstract

**Introduction:**

Parents whom adolescents disclose sexual abuse face both a personal traumatic experience, and the need to support their child who is going through a grueling period and needs them. Many quantitative studies exploring the psychological impact of disclosure on parents have been conducted, but few have used qualitative methods. The objective of this study is to explore parents’ experiences of their adolescent’s disclosure of sexual abuse during psychiatric care, identify the possible beneficial factors and shortcomings, share this knowledge, and improve interventions for these families.

**Methods:**

We conducted semi structured interviews with parents whose children disclosed sexual abuse during their psychiatric care in Paris and analyzed these interviews using a phenomenological framework (interpretative phenomenological analysis).

**Results:**

This study analyzed 13 semi structured interviews of 9 mothers and 4 fathers whose children were then aged 14 to 17 years. Qualitative analysis uncovered three themes: (1) Parents: alerts and search for support; (2) Between parents and adolescents: a disruption in relationships linked to the disclosure and its legal consequences; (3) Disclosure at the family level: the possible reactivation of a traumatic past and the search for a new equilibrium.

**Conclusions:**

Considering the parental experience is essential in caring for adolescent patients after they disclose sexual abuse. The need for parental or family psychological support should be systematically assessed. Possible resurgence of parental trauma requires psychiatrists’ careful consideration.

**Supplementary Information:**

The online version contains supplementary material available at 10.1186/s12888-023-05410-7.

## Background

Sexual abuse is defined by the World Health Organization (WHO) as “any sexual act, attempt to obtain a sexual act, unwanted sexual comments or advances, or acts to traffic or otherwise directed against a person’s sexuality using coercion, by any person regardless of their relationship to the victim, in any setting, including but not limited to home and work [1].“ One woman in 5 and one man in 13 reports experiencing sexual abuse in childhood [2], and one person in 10 reports being subjected to incest [3].

The psychological consequences of these traumas are well known and documented today [4]: individuals subjected to Child Sexual Abuse (CSA) are at higher risk of developing depressive disorders, post-traumatic stress disorder, schizophrenic disorders, or bipolar disorders, as well as of attempting suicide [5–7]. The prevalence of a history of experiencing CSA in persons seen for psychiatric care (adult, adolescent, or child) is higher than in the general population [8]. Moreover, the somatic consequences currently identified of a personal history of experiencing CSA include, for example, increased functional intestinal disorders and nonspecific chronic pain [9].

Despite these multiple severe complications and their elevated prevalence, only 20–30% of these individuals who have been subjected to CSA confide in a health professional or a community organization. Disclosure, when it does occur, takes place on average more than 12 years afterwards [3]. This disclosure is a necessary prerequisite to appropriate psychological care, which has positive consequences on mental health, by reducing post-traumatic symptoms [10, 11] and neurobiological damage (e.g., serotonin abnormalities and impairment of the adrenergic axis) [12]. It can also make it possible to avoid the phenomena of continued abuse (repetition of the situation of CSA) [13] as well as protecting other potential targets (by identifying the aggressor). Inversely, disclosure can have negative consequences if the persons told lack empathy, question or disbelieve the patient’s word, or make her or him feel guilty [14].

Parents of adolescents who disclose CSA are at the heart of this disclosure process. There is some evidence that they experience an important stress [15] and experience lower feeling of self-efficacy [16], which affects their relation with their child. Their reaction influences their child’s course after the disclosure [17–19]. For example, a prospective quantitative study of 30 children aged from 7 to 12 years and their parents showed that the children who expected a negative reaction by their parents tended to delay divulging the violence and that their apprehension was justified because up to 65% of parents showed themselves unsupportive after the revelation. The parental reaction was all the more negative (non-supportive, rejecting) when the abuser was someone the family knew and when the abuse was severe [20, 21]. The parents’ initial reaction may play a crucial role in the likelihood that the children blame themselves — a reaction that frequently leads to self-harming thoughts and behavior [14]. Nonetheless, these results must be carefully interpreted, because the evaluation of parental reactions by quantitative studies is not adequately standardized, that is, they do not use standardized scales. The association between the type of parental reaction and the child’s course afterwards is not clear [22].

Moreover, these quantitative studies are limited by the frequent differences in the way they measure the concept of parental support but also by their research methods, which cannot examine the full complexity of the parental experience [22]. Qualitative studies can be more appropriate for approaching the parents’ experience of this disclosure and for understanding their lived experience, the meaning they give to their own attitude and to what their child has endured. The experience of parents — a topic to which researchers have long paid little attention — is now being increasingly considered, by studying their point of view directly and no longer only that of the child. A better understanding of the parental experience is a pathway to improving our support of these parents during this period when their child needs their support. Few qualitative studies have examined the experience of parents of adolescents disclosing CSA. An existential-phenomenological study focused on fathers and underlied their supporting role, and the need for psychological help [23]. Another study, most important, highlighted the importance of the parents’ experience, showed how they tried to make sense of the abuse in retrospect, and how their identity as protectors was challenged [22]. These studies provided a crucial qualitative foundation on the topic. However, there is a need for additional research exploring parents’ experiences, particularly in a different country with diverse backgrounds. Furthermore, there is a requirement for a more in-depth explanation of the various relational mechanisms at play between parents and their children who have disclosed CSA. We have attempted to explore this subject. To the best of our knowledge, there hasn’t been a qualitative study conducted in France regarding the experiences of parents after their adolescent children have disclosed CSA.

The objective of this qualitative study is to examine the experience of parents whose adolescents have disclosed to a health professional during their psychiatric care that they had been subjected to CSA. In this research, we study disclosure of sexual abuse through both the experience at the moment of disclosure and its longer term consequences on family. The purpose of this study is to use these parents’ experiences to offer perspectives for treatment to improve the support of these families. As the importance of interventions for supporting caregivers has been shown before [24].

## Methods

### Type of study

This exploratory qualitative study is based on interpretative phenomenological analysis (IPA), which is based on an iterative, inductive process [25]. The objective of IPA is to discover in a natural setting how subjects experience and give meaning to a phenomenon, by studying what they say about it [26]. IPA is a recognized method of discourse analysis, especially in health psychology. The researchers considered their own sources of bias and prior assumptions, including knowledge and experience gained from working in adolescent mental health services (EC, PM, RR, SBG, MRM) and conducting research into young people’s mental health (EC, RR, SBG, MRM). The epistemological standpoint of this study is interpretativist. Knowledge is perceived as subjective and contextual. Significance is attributed to individual perspectives and how individuals make sense of their experiences. It is also worth noting that the researchers who participated in this study have diverse backgrounds, especially systemic family therapy, but also neurobiological psychiatry, and transcultural psychiatry.

The French institute of medical research and health’s (INSERM) ethics assessment committee approved it (IRB00003888) in April 2021. We followed the guidelines for ensuring rigor and reflexivity in qualitative research, in the second part of the Qualitative Research Methods in Mental Health and Psychotherapy book (Method, Interpretative Phenomenological analysis), by Harper ad Thompson [25, 27], and used the COREQ (COnsolidated criteria for REported Qualitative research) checklist to report qualitative research [28].

### Participants and sampling

The participants were recruited by a female psychiatrist, EC, in a center of adolescent medicine and psychiatry (*Maison des Adolescents*) in Paris, France. These multidisciplinary integrated youth health care centers receive and evaluate for adolescents and their families, providing them with medical, psychological, socio-educational, educational, and legal services [29]. We recruited the parents of adolescents seen for outpatient therapy or in either our day hospital or the inpatient psychiatric unit and who had learned during this care that their adolescent had disclosed to professionals that she or he had been subjected to CSA. When an adolescent divulges such abuse for the first time as part of an interview with a care provider (child psychiatrist, psychologist, pediatrician, or nurse), this professional is required by law to inform the minor’s legal representatives of these facts [30]. The provider — working with the teen — thus systematically organizes an interview to inform the parents and discuss this information with them. As specified in the French public health code, this professional must also submit a report to inform the appropriate child protective authorities, so that they can assess the minor’s situation and determine the protective actions and assistance available for the minor and the family, beyond the psychiatric/psychological care. This report also marks the official recognition of the adolescent’s disclosure [31]. It can thus lead to the filing of official charges — or not, as the teen wishes. Families and adolescents are systematically offered a meeting with the department’s social worker, who can explain to them in greater detail the issues related to the reporting and support them through the social and legal procedures. The medical and psychological care continues and is adapted according to the clinical course and the teen’s needs.

Inclusion took place from October 2021 through July 2022. The participants were recruited by purposive sampling [32], that is, the researcher selected the subjects likely to furnish the most information possible about the phenomenon being studied. We continued including parents until we reached data saturation. Following the principles of maximum variation, we recruited parents whose participating adolescents differed most from one another in terms of age, sex, sex of the abuser, the type of CSA (intra- or extra-familial), the teen’s age at the time the CSA occurred, psychiatric or physical symptoms, and the family’s socio-cultural status. The analysis took place at the same time as the data collection, which continued for as long as the analysis of material continued to provide new and useful information [33].

The inclusion criterion required that the parents had an adolescent, male or female, aged from 11 to 20 years who was receiving ongoing child psychiatry care and had disclosed to a health professional that he or she had been subjected to CSA. Parents were excluded when they were accused of CSA by their child or if they presented acute psychological distress requiring that care be prioritized over research.

### Data collection

Parents received oral and written information about the study. If they were interested, they were offered an interview so that we could explain the study organization and the research procedures, describing all relevant information, and then obtain their oral and written consent. The researcher collected social and demographic data with a questionnaire. We performed thirteen individual interviews, including four fathers and nine mothers— the parents of eight adolescent girls and one boy (Table 1). Five parents refused to participate in the research interview without explaining why.

**Table 1:** Description of participants

	Relation to child	Child’s age at disclosure	Site of care at disclosure	Official report*	Filing of charges*	Relation of the adolescent to the abuser	Personal or family history of sexual abuse
**P1**	Mother of Audrey	16 years	Inpatient hospitalization	Yes	No	Familial (cousin)	Yes
**P2**	Father of Audrey	16 years	Inpatient hospitalization	Yes	No	Familial (cousin)	No
**P3**	Mother of Anaïs	15 years	Inpatient hospitalization	Yes	No	Familial (brother)	Yes
**P4**	Mother of Géraldine	14 years	Outpatient	Yes	No	Non-familial (friend of the mother)	Yes
**P5**	Mother of Chloé	15 years	Outpatient	Yes	No	Familial (half-brother)	Yes
**P6**	Mother of Juliette	14 years	Inpatient hospitalization	Yes	No	Non-familial (known neighbor)	No
**P7**	Father of Juliette	14 years	Inpatient hospitalization	Yes	No	Non-familial (known neighbor)	Yes
**P8**	Mother of Rose	15 years	Outpatient	Yes	No	Non-familial (unknown)	No
**P9**	Father of Rose	15 years	Outpatient	Yes	No	Non-familial (unknown)	No
**P10**	Mother of Noémie	16 years	Outpatient	Yes	No	Non-familial (high school student)	No
**P11**	Father of Noémie	16 years	Outpatient	Yes	No	Non-familial (high school student)	No
**P12**	Mother of Clément	17 years	Inpatient hospitalization	Yes	Yes	Familial (mother’s cousin)	No
**P13**	Mother of Blanche	15 years	Outpatient	Yes	Yes	Non-familial (Middle school students)	No

* A report to the child protection authorities must be made when a minor reports a dangerous situation, such as sexual abuse

* Charges can be submitted to the legal authorities by the parents following a disclosure by minor

The data were collected in the work center by a female psychiatrist, M.D. (EC) and a male resident in psychiatry, M.D. (PM), both trained in qualitative research. Interviewers did not have professional relations with the parents interviewed. Each parent was interviewed alone, in a face-to-face semistructured interview of around an hour. A semi-structured guide (see Appendix 1) for each group was developed based on the authors’ clinical experience and research expertise in the fields of adolescence, family, child sexual abuse and qualitative methodology. Questions were evaluated by clinical experts, piloted with parents, and revised accordingly. The interview guide explored the following topics: the context of the adolescent’s care, the context and experience of the disclosure, the individual reactions that parents identified both in themselves and their children, and any modifications of family relationships after the disclosure. The researcher kept in mind a list of themes that had to be covered, but the discussion nonetheless remained very open, to make it possible to gather richer data about each experience. The audio content of the interviews was recorded with a dictaphone; PM later transcribed it and pseudonymized it.

### Method of analysis

The interviews were analysed by applying Interpretative Phenomenological Analysis (IPA) method.

We used the following analytic procedure: the interviews were read several times; the researcher (PM) annotated the text of each interview with initial comments. These comments were then grouped into themes that succinctly summarized their essential characteristics. Connections between the themes were then drawn until we obtained a coherent thematic organization of the interview. Meta-connections between interviews were then identified, to determine a set of meta-themes describing all of these narratives. Each meta-theme was linked to the underlying theme, itself linked to the original annotations and interview extracts. The meta-themes were then explained in a written report that underlined the themes, accompanied by interview extracts. At each stage, the researcher verified in the material that the groupings were consistent. The researcher thus went continuously back and forth between the analytic data and the source material. Each interview was analyzed immediately after it occurred. This enabled us to reorient the questions raised in the interviews that followed to obtain the richest material possible. These interviews were triangulated by different researchers (EC, PM, RR, SG, and MRM), as well as in a seminar with a group of researchers specialized in the IPA qualitative method. Alternative interpretations were considered and discussed until a consensus on the interpretation of patterns in the data was reached. Triangulation made it possible to ensure that the themes identified most accurately reflected the data. Discussing, clarifying, and, if necessary, modifying the themes helped to improve the study’s validity and to limit the interpretative biases specific to the IPA [34]. The analysis was assisted by NVIVO 11© software.

## Results

The data analysis allowed us to identify nine themes organized in three meta-themes: (1) Parents: alerts and search for support; (2) between parents and adolescents: a disruption in relationships due to the disclosure and its legal consequences; (3) disclosure at the level of the family: the possible reactivation of a traumatic past and the search for a new equilibrium (See Table 2).

**Table 2:** Summary of themes

Meta-themes	Themes	Description
1. Parents: alerts and search for support	1.1. Before disclosure: what alerted parents and how they sought care for their child	Diverse signs of distress in adolescents identified by parents that led to a consultation with a specialist.
	1.2. During disclosure: emotional reactions including shock, guilt, anger, and empathy	A broad range of emotions felt by parents at the moment of the disclosure.
	1.3. After disclosure: parents seeking individual support and guidance in parenting	Search for support of distressed parents after the disclosure.
2. **Between parents and adolescents: a disruption in relationships linked to the disclosure and its legal consequences**	2.1. Disturbances in the relationship equilibrium and in parent-child communication	After the disclosure, an important impact on parent-child communication and the dynamics of their relationship.
	2.2. Legal proceedings: relief, doubt, and rejection	Between the mandatory reporting and possibility of legal charges, parents feel supported, but also judged, or even rejected.
3. **Disclosure at the family level: possible reactivation of a traumatic past and the search for a new equilibrium**	3.1. Reactivation of old family trauma	Memories of sexual abuse in parents or their family revived by the disclosure.
	3.2. Search for a new equilibrium	With time, the reorganization of family functioning.

### Parents: alerts and search for support

This first meta-theme describes how parents were alerted by their teens’ signs of distress, the emotional reactions associated with the disclosure, and their search for individual support and for guidance in their parenting.

#### Before disclosure: what alerted parents and how they sought care for their child

Signs of ill-being alerted the parents and led them to mobilize to find appropriate care for their child. The participants reported perceiving their children’s ill-being because of their anxiety, sadness, social isolation, or cutting and other forms of self-harm. Risk behaviors also frequently warned parents about the existence of a problem in their adolescent:

*“It was the first time that we’d seen her in this state [of drunkenness], a child who hadn’t ever drunk! Well, an endangerment, in fact, an endangerment, but dreadful. She vomited all night (P10, 16) (Parent, child’s age at disclosure).”*

These parents perceived that the child’s distress might affect her relation to school, with either absenteeism, or a degradation in her grades.

Parents also worried about changes in the child’s relationships with their peers or with themselves.

*“But in fact I rapidly understood that at a physical level she couldn’t stand for me to touch her, for me to come near. She hates my smell, my breath, in fact, even if she doesn’t smell it, she smells it very well. That is, there’s a real rejection (P4, 14).”*

The onset of conflicts or ruptures in communication also alerted parents about the child’s distress:

*“She didn’t want my help anymore in her work, which I’d always done before, that is, when help was asked for, there was no problem. Now, ‘no’ was systematic, ‘I don’t need it.‘ The situation, the communication between us deteriorated progressively throughout tenth grade (school year 10). … Our relationship with Rose was entirely made up of conflict (P8, 15).”*

Several parents shared the impression that they no longer “recognized” their child and worried that something that would hurt her might be hidden from them.

Thus, the feeling of reaching one’s limits for understanding the child’s difficulties and being incapable of providing help led families to seek professional help and begin specialized care The observation of psychological distress and inability to find a solution led parents to look for help outside the family:

*“I said [to my daughter]: ‘I don’t know what’s going on, but in any case, the three of us can’t solve it, because we’ve tried and failed… (P10, 16)”*.

#### During disclosure: emotional reactions including shock, guilt, anger, and empathy

The start of psychiatric care for the adolescent provided the occasion to reveal the CSA, past or present. After disclosure to the physician, the parent was informed either directly by the adolescent (for six parents), or by the physician (seven parents).

Parents described their shock, their stupefaction at the moment of the disclosure, using numerous metaphors to underline the intensity:

*“I swear, I was thunderstruck … It’s a tsunami in a parent’s face, a thing like that (P1, 16).“ “I fell off a cliff. … I was stunned. … It was as someone had cut off my legs (P5, 15).”*

Sometimes, related to this shock, several parents could not help but express doubts about the veracity of the facts reported:

*“We [father and mother] are flabbergasted, especially me. I even doubted it because, as there was some tension, I didn’t know how much truth there was in what she said (P11, 16)”*.

The primary affect, dominating all others in the entire set of interviews in this study, remained guilt. To not have seen, not have known how to listen to the child, not have known how to protect her: parents reproached themselves strongly:

*“It was pretty hard to take that in. To take it in because you have a feeling of failure… of not having seen that she wasn’t well. Because if the sexual assault dated to the 7th grade (when she was 12), that means that there were signs, that we didn’t see them (P10, 16).”*

They often had the impression that they had failed to fulfill their role as parents.

It also happened that parents felt responsible for the child knowing the abuser. This was the case here, where the mother’s partner was the abuser named by her daughter. The guilt was thus intense:

*“No one is going to feel sorry for me because I’m the mother of a child who was abused*. *Because in fact no. There is obviously something wrong because for a parent to put [the child] in a relation with an abuser, well finally, that’s it. That can only happen through school or the family. That’s clear. But [we’re] the link between the abuser and the child, that is, the victim (P4, 14).”*

Parents also express great anger toward themselves, but also towards the abuser, voicing ideas of vengeance:

*“Knowing that a boy abused my daughter, what should I have done? … Go to the school and break this little young man’s legs because I was furious? … I felt angry (P11, 16).”*

Finally parents proclaimed especially their empathy towards their child:

*“[We said to her] that we really felt awful for her and that now that she had talked about it, that was really good, and that was going to be able to help her, truly (P6, 14).”*

But the parent’s empathy could also be expressed toward the juvenile aggressor:

*“For us, somewhere, he was also a victim of this, he will also have to take himself in hand to heal in fact, that he also needed to heal from that (P1, 16).”*

#### After disclosure: parents seeking individual support and guidance in parenting

After the disclosure of CSA, parents were very affected emotionally and sometimes reported a need for psychological support for themselves. The child’s psychiatric care means that the parents met regularly with the doctor to discuss the course and management. Parents were able to feel institutional support for themselves, from their child’s psychiatrist, in relation to the difficulties they were experiencing with the child. This association was important for them:

*“And from the beginning of therapy, therefore from before the disclosure until today, we always had this link with Madame X, we could always call her, we could always see her, for every question, every problem (P7, 14).”*

Inversely, some parents reported that inadequate time was allocated to them to allow them to discuss their concerns and ask their questions after the disclosure. Beyond wanting to express their personal feelings as parents, they talked about their need for guidance for the family in this exceptional situation:

*“How do we tell the other children? Do we have to tell them? At what age? How do we explain family secrets? …. That’s what I would have needed (P1, 16).”*

Several parents expressed the need for interviews for themselves or individual therapy, which they had missed:

*“The management isn’t adequate, really. It’s not adequate. … it would be good if there was more systematic support for parents (P7).”*

They had doubts about their parenting skills and would have liked to be guided individually in their decision making:

*“I’ve made an appointment with a shrink in private practice to be able to discuss some things, because in fact we don’t know if we’re doing things right or not. There’s nothing offered for parents for that (P6, 14).”*

### Between parents and adolescents: a disruption in relationships linked to the disclosure and its legal consequences

This meta-theme deals with the effects of disclosure on the dynamics of the parent-teen relationship, as well as the legal procedures and their effects on this relationship.

#### Disturbances in the relationship equilibrium and in parent-child communication

After the disclosure, the balance in the parent-child relationship was disturbed, and their communication could be impeded, even interrupted.

The difficulty in adjusting to the shock of the disclosure could come from the parents rather than the adolescent:

*“Up until yesterday evening, when we talked a little, our relationship had not actually improved… I realized that I was not ready, in fact, and that she certainly expected that (P1, 16).”*

Or the distancing could come from the adolescent:

*“But it’s very hard not to be able take her in my arms and give her big kisses. Anyway, it’s hard to be rejected all the time; it’s complicated (P4, 14).”*

The important increase in parental worries after the disclosure sometimes impeded the pursuit of a more peaceful parent-child relationship:

*“In any case, that created a relationship strongly marked by all that; that is; I worried very much… I also pointed out to her that she was sick, that she’s ‘not well’ in quotation marks. And as a result there’s still a kind of anxiety still there between us (P6, 14).”*

Communication in the family was also questioned according to whether it was or was not possible to talk about the facts disclosed. The subject might be experienced as too difficult to talk about or needing to be avoided completely, so that it’s not thought about anymore and the family can “turn the page”:

*“I haven’t talked about it because for me it’s finished, over and done with. … The page is turned (P3, 15).”*

Another choice parents could make was to voluntarily avoid the subject of CSA, with the idea of leaving the child free to raise it:

*“We don’t talk about, we don’t talk about [her brother]. If Chloé wants to talk about it, we will, we’re here (P5, 15).”*

#### Legal proceedings: relief, doubt, and rejection

Some parents experienced the reports written by the health professional after the adolescent’s disclosure as a relief and a recognition of the child’s status as the victim of a crime:

*“There was a lot of gratitude and recognition that it was done, that it was put down as non-negotiable in fact. It’s like that and it was so good (P1, 16).”*

But the report to the child protective authorities was sometimes experienced as a violent procedure difficult to accept, in particular in situations disclosing intrafamily sexual abuse, as for this mother whose daughter reported incest with her oldest son:

*“When they [the doctors] talked to us about an investigation, the police, there it wasn’t the sky but the universe that crashed down on my head (P3, 15).”*

The consequences of reporting was sometimes very frightening, especially because of how slowly legal procedures move. Some parents explained to us that more than a year after the disclosure and the report, they were still awaiting a response by the child protective department or the court system:

*“We’ve always got a knot in the stomach about what’s going to happen. … But in the end, we’ve waited days, weeks, months, and we’re always afraid at every moment that the doorbell will ring, after we’ve resumed our life (P3, 15).”*

This late legal response can make the family work still more complex, freezing the conflicting relations and postponing the possibility of work to find a new family equilibrium.

As to filing criminal complaints, that happened only for one study participant. Most refused to do it because of uncertainty about the expected effects.

*“In fact therapeutically, I’m sure that it’s important to do it, … but after you also have to balance: what is going to happen and is that not going to take away everything positive that’s happened? (P1).”*

But they based their decision most often on the choice of their child, who did not want to bring charges:

*“She has repeatedly made it clear that for the moment she absolutely does not want to file charges and does not want us to do it. … We are not going to go against her wishes for this, because we think she’s mature enough to make this decision (P6, 14).”*

The parents therefore described living through very different experiences of this reporting process, between those who felt supported and relieved by this procedure, and those who felt hurt, living through an apparently interminable anxiety-ridden situation. Parents might understand filing charges as an important process, but some obstacles overcame this, especially the child’s opposition.

### Disclosure at the family level: possible reactivation of a traumatic past and the search for a new equilibrium

This meta-theme concerns the memories that the disclosure calls up in the parents about their own history, changes in the family dynamics, and finally the perspective of a new family equilibrium.

#### Reactivation of old family trauma

Five participants spontaneously mentioned their own history of CSA, linking what they had experienced to what their child had reported.

One mother, for example, recognized feeling that she confused the attack on her daughter with the one against her some 30 years ago and worried she was reacting as a child exposed to abuse rather than as the mother of such a child.

*“I was abused at the same age for years, and I’d forgotten it. It took me 31 years, with lots of symptoms all my life … for these memories to return. … I feel that I sometimes forget that it isn’t me involved. It’s rather in the other direction, I forget that it’s Audrey who’s involved. And so I feel that my reaction is not that of Audrey’s mother but of the little girl I was (P1, 16).”*

The difficulty in differentiating herself from her own daughter, when it comes to this trauma resembling her own, shows how this mother’s parenting capacity is undermined by this repetition of her own history in her daughter’s life.

One adolescent’s action distancing herself from a parent also recalled the reaction her mother herself had had in childhood after the abuse:

*“Finally I understood, as I had also experienced CSA when I was a small child, and thus it’s by the similarities to my reactions to my parents … finally I could not approach them. It was a physical rejection, a rejection I couldn’t control (P4, 14).”*

Some parents reported a repetition of this violence across generations, even though they themselves had not directly experienced this abuse. When his daughter disclosed the CSA she was subjected to, her father remembered the story of his mother, who had revealed that she had been abused by her own father. This father’s questions then confronted him with the taboo a family can create around these questions:

*“I had asked too many questions and besides, yes I was the one, according to my sisters, who was stirring things up… My parents were quite old and I spoiled their end of life by stirring up shit a little (P7, 14).”*

#### Search for a new equilibrium

After the disclosure, intrafamily relationships moved from their temporary fragility to a reorganization that could, according to some participants, help improve communication and reinforce the weakened family structure.

After the disclosure, several parents felt that the equilibrium in their family had become fragile:

*“It’s very complex in fact, to not trivialize it, to give it enough importance, and at the same time, to not drag the entire family dynamic into it*. *Because there are also other children (P2, 16).”*

But later on, after the disclosure, a new equilibrium was sought. Parents mentioned the need for family care for this. How can family life be maintained? How can we talk as a family? How can the siblings be supported?

*“We have a family, it’s a family system, we take care of the child, it is important to take into consideration the repercussions of this announcement within the immediate family… In fact, I think I would have needed a space to talk with a family therapist so that we could talk about it as a family (P1, 16).” “We also worry about the little brother; he can’t be overlooked. There we were advised, we took the step, we started a work of family therapy, to give him a space to talk too (P6, 14).”*

The disclosure could be recognized afterwards as propitious to change in the relationship:

*“She didn’t have to weigh her words, to pay attention to what not to say. Now it’s opened up the relationship some. … There’s movement, that’s what I feel, it’s that there’s movement; therefore it’s positive if there’s movement (P1, 16).”*

Finally the dynamics often moved toward rapprochement and increased support between the family members:

*“We talk much more today than before with my daughter (P7, 14).“ “Yes I’ve got my daughter back. … Disclosure led to a real rapprochement (p8, 15).”*

Moreover, support between the couple grew, even during these trials:

*“My husband and I supported each other (P3, 15).”*

## Discussion

In this study, we interviewed 13 parents who had learned that their adolescent had disclosed having been subjected to sexual abuse within or outside their family to a health professional seeing the child for psychiatric/psychological care.

Our analysis shows that the disclosure was not seen as an event isolated in time, but as a dynamic and changing process with several phases. The interviews enabled us to describe these different phases at the level of the parents, of the parent-child relationship, and finally of the family. It was the parents who first became aware of their child’s state of psychological distress, which led first to the start of care and then to the disclosure. This in turn led to a dislocation in the parent-teen relationship linked to the content of this disclosure and its traumatic dimension but also due to the important impact of the legal process on the family dynamics. Finally, some families experienced a particular shock because of the reactivation of old family trauma on the occasion of the adolescent’s disclosure. The question of words, of talking, appears in the foreground: how to talk to each other, whether to talk about it with the teenager or to avoid the subject of sexual violence, how to talk with the rest of the family, especially the siblings. Families could progressively find a new equilibrium, sometimes by the parents seeking guidance, sometimes by family therapy.

These results are near from the similar qualitative studies on these subjects [22, 23], underlying the difficulty for the families to cope with the shock of the disclosure, but also to support the legal procedures, and at the end highlight the importance of the involvement of the parents in the child therapy. Our study reinforces the importance of the capacity of the institutions to receive and treat the parental experience of this disclosure.

### The reactivation of intergenerational family trauma

Four mothers in our study spontaneously reported that they had experienced sexual abuse during their childhood or adolescence, some by an individual outside their family and some by family members. For one father, his daughter’s disclosure reminded him of the intrafamilial CSA his mother had experienced and another was reminded of his family’s lax incest taboo. These different parents noted that their child’s disclosure had made these memories re-emerge by bringing back to the foreground this personal or familial traumatic experience.

A phenomenon of the repetition of trauma across generations thus appears. We will approach this particular point through the concept of the “intergenerational cycle of sexual victimization in childhood.“ This concept, developed by Baril and Tourigny, defines the existence of CSA of both the parent and child, in situations where this parent was not the abuser [35]. Ever more studies of this topic have recognized that the presence of a history of sexual abuse in a parent is a risk factor for repetition for the next generation [36]. The risk of this repetition is greater when the parent has not worked on this trauma [37].

Several hypotheses have been set forth to explain this association. First, one of the main explanatory themes is the silence around traumatic events, which leads to feelings of solitude, isolation, and loss of confidence [38]. The frequency and quality of parent-child communication appears to be a protective factor against CSA.

Other authors have suggested that parents abused in childhood may be spontaneously attracted to partners with a type of personality similar to that of their former abusers and who may therefore repeat the act of abuse toward their child [39]. That is, there may exist insecure attachment patterns in survivors of CSA that may affect their relationships in adulthood [40]. This hypothesis is specifically echoed in the situation of one mother participating in our study, who had been sexually abused in childhood and was in a relationship with a man who abused her daughter. Mothers abused in childhood are more likely to report difficulties in parenting, together with other difficulties secondary to their past of CSA, but still present in adulthood [35]. This is particularly visible with the example of the mother in our study that is in great difficulty to differentiate her own experience from her child’s one. She feels unable to act as a mother in this very moment. These supplementary difficulties associated with a history of past abuse should be looked for, together with depression [41], anxiety [42], low confidence in parenting skills [41], poor quality of partner relationship [43], and intimate partner violence [44]. Our qualitative study, by focusing on these parents’ lived experiences, allows us to better understand these associations between the intergenerational history of CSA and difficulties in parenting.

Complex trauma is one way to conceptualize the diversity of sequelae cited and documented from parental CSA. Complex trauma corresponds to a traumatic event in a relationship — prolonged, repeated, occurring during a developmental period, as is the case in several CSA situations [45]. In addition to the standard events related to PTSD, it leads to complex developmental consequences in childhood and adulthood [46]. A proposal for an evaluation and care for the parents themselves and for the family as a whole thus appears indicated in situations where a child reveals CSA. These results also show that the patients who have experienced CSA require particular attention from therapists when they become parents, to support them in this new role, now that it has been shown that their children are at a higher risk of becoming CSA in turn.

### Essential parental and family support

Our results showed that within the process of disclosure, families went through relationship disruptions and that parents formulated their need for specific support. Our data concord with the results of numerous studies that have set forth the effects — possibly traumatic — of disclosure on parents [47] and a substantial effect on their parenting abilities [48]. A qualitative study used questionnaires to focus specifically on parents’ needs after disclosure [49] and showed, consistently with our findings, a need for help in managing the child’s behavior and support for overcoming this challenge.

A recent qualitative study in Ireland explored the experience of both mothers and fathers, as we did, but unlike the previous studies that focused only on mothers’ experiences. This study questioned 10 mothers and 4 fathers. It found as we did that parents were challenged in this situation, with a trend toward hypervigilance and overprotecting the child [22]. Moreover, it showed that parents’ feelings of shame and self-reproach could lead them to distance themselves from the trauma linked to this disclosure, which can create a barrier to access to care for their child [50]. These too are elements we found in our results.

Support to parents is essential in the post-disclosure period. The entire process of the disclosure must be considered together: before, during, and after. It has been shown that parents able to support their child after the disclosure — that is, to recognize the child’s status as abused, listen to her emotions, support and accompany her through medical/psychiatric care and the legal steps that may follow — is one of the most important factors promoting a favorable outcome for the child [51]. Moreover, after the child’s disclosure, psychological distress appears to be greater among parents reporting a history of CSA. This findings suggest that these parents have specific clinical needs [52, 53]. Moreover, a sexual abuse survivor faced with his or her child’s disclosure may be triggered to disclose his or her own history. It is therefore necessary to: evaluate parents’ emotional reactions and possible psychological difficulties as part of managing adolescents’ disclosure of CSA and offer an intervention adapted to the needs of each member of the family. This attention towards parents can involve care provided directly to a parent whose past sexual trauma can resurface on this occasion [37], but also care for the family as a whole, as the participants in our study mentioned. A multigeneration study showed the interest of prevention programs for families in which a parent was sexually abused in childhood, to limit the risk of intergenerational repetition [54].

Recent decades have seen numerous quantitative studies of treatments involving simultaneously parents and the child who disclosed sexual abuse. Several types of therapies have been successfully tested, such as “filial therapy” [55], structured therapeutic games [56], and child-parent relationship therapy [57]. The Intergenerational Trauma Treatment Model program is one of the rare intervention programs with the objective of improving parents’ ability to respond to the needs of their child who has been subjected to trauma, such as CSA, while simultaneously considering the effects of the parents’ own CSA [58]. In addition to interventions focused on the child, this program includes interventions based on a cognitive-behavioral approach for parents who have had a traumatic experience. These aim to diminish the symptoms of post-traumatic stress, regulate emotions and behaviors, improve attachment relationships, and increase parental skills/competence in empathetic response to their child’s needs [59].These varied therapies have in common the inclusion of both parent and child and strongly support the interest of an approach that includes parents in the therapy. A meta-analysis including several studies with control groups confirmed the advantage of a therapy including parents over therapy for the child alone [60].

The limitation of these approaches is their restriction to the parent/child subsystem, excluding the other family subsystems. The parents in our study raised the question of the impact of the disclosure on the siblings, and the literature on this particular point is sparse. We thus suggest that research in this domain should be pursued, examining the experiences of the siblings of children and adolescents who have disclosed their CSA.

The participants in our study also raised the question of the interest of specialized interviews with an expert in the topic of CSA after the disclosure. This question has been approached in an article suggesting the need for the recognition of a subspecialty in CSA for mental health professionals [61]. This is indeed a particular facet of care that requires that professionals have specific competences and skills. Thus, for some specific complex situations, professionals specialized in the issue of CSA could see the family and the child. This might increase the confidence of the families and the effectiveness of the care. Nonetheless, this referral should be ad hoc, specific, and second-line, to avoid reducing the management of the adolescent and the family solely to this issue.

### Strengths and limitations

This cross-sectional study examined the parents’ experience at a particular moment. It would be interesting to study its evolutions and changes in a longitudinal study, so that parents’ experiences could be assessed after time has passed. It could allow us to examine if their needs remain the same, how time affects their feelings and memories of this experience. So it could change the point of view of professionals on the particular moment of the disclosure, knowing better what happens next for the parents.

The single-center character of our research implies that our results may be influenced by the specific organization of our child psychiatry department. Our questioning of both mothers and fathers of adolescents seen in different units, by different psychiatrists, and at different intervals from the disclosure allowed us to limit this bias. The protocols and support following an adolescent’s disclosure can vary from one department to another, from one specialty to another, and from one region or country to another. In the future, it would be worthwhile to conduct similar qualitative studies in other departments and other countries.

## Conclusion

This study shows the importance of taking parents’ experiences and their needs for support into account after the disclosure of CSA experienced by their adolescent. In this process marked by individual and relational disruption, parents were able to describe the essential requirements for support for themselves and for the entire family. The disclosure of CSA by an adolescent can reactivate traumatic memories in their parents. It is therefore useful to explore and enable parents or other family members to express these histories.

After the disclosure of sexual abuse by an adolescent, we recommend a systematic evaluation of the need for an interview or even individual parental care and family care, together with the adolescent’s treatment.

### Important points

- Parents report a disruption in the relationship with their child and the fragilization of family relationships after the disclosure of the sexual abuse of their adolescent.
- Some parents of children divulging sexual abuse report a reactivation of old intergenerational sexual trauma.
- After the disclosure, parents feel a need for individual support and specific guidance in parenting.
- Parents point to the need for aid at the level of the family, to take the siblings into account.
- We advise an evaluation of the parents’ needs for individual care and of family therapy in cases of the disclosure of sexual abuse in children.

### Electronic supplementary material

Below is the link to the electronic supplementary material.

Supplementary Material 1: Semi-structured interview guide

## Data Availability

The raw data supporting the conclusions of this article will be made available by the authors, without undue reservation. Contact: philippe.mauny@gmail.com.

## List of Abbreviations

COREQ: COnsolidated criteria for REported Qualitative research
CSA: Child sexual abuse
IPA: Interpretative phenomenological analysis
ITTM: Intergenerational Trauma Treatment Model
WHO: World Health Organization
