# Supplementary material for: How parents experience their adolescent’s disclosure of previous sexual abuse: a qualitative study
Source: BMC Psychiatry. 2023 Dec 6;23:916. doi: 10.1186/s12888-023-05410-7 (PMC10698959; doi:10.1186/s12888-023-05410-7)
Supplement: Supplementary file 1 — Supplementary Material 1: Semi-structured interview guide [file 12888_2023_5410_MOESM1_ESM.docx]

Semi-structured interview guide

- Q1. For what reason was your child receiving outpatient or inpatient psychiatric care?
- Q2. How did you learn that your child had been sexually abused?
- Q3. How did you feel after you learned of this abuse?
- Q4. What did you think of the support that your child — and you yourself — received after this disclosure?
- Q5. How did you experience the legal procedure, and the filing of the complaint? Was there one?
- Q6. What consequences did these disclosures have on your relationship with your child?
- Q7. More generally, what were consequences of these disclosures on your child's life, and on yours? Were they, on the whole, positive or negative?
